# Accurate and efficient P-values for rank-based independence tests with clustered data using a saddlepoint approximation

**DOI:** 10.1038/s41598-025-26728-0

**Published:** 2025-11-25

**Authors:** Haidy A. Newer

**Affiliations:** Department of Mathematics, Faculty of Education, Ain Shams University, Cairo, 11511 Egypt

**Keywords:** Saddlepoint approximation, Rank-based tests, Clustered data, Independence testing, Permutation tests, Block urn design, Non-parametric statistics, Tied ranks, Computational biology and bioinformatics, Health care, Mathematics and computing, Medical research

## Abstract

Accurate statistical inference for clustered data—common in multi-center clinical trials and longitudinal studies—poses significant challenges due to within-cluster correlation. Rank-based tests like the logrank, Wilcoxon, and Datta-Satten are valued for robustness but often suffer inflated Type I error rates under standard asymptotic approximations. While exact permutation tests offer theoretical accuracy, they are computationally impractical for large datasets, highlighting a methodological gap. This paper proposes a double saddlepoint approximation framework to deliver accurate p-values and confidence intervals for a wide class of rank-based tests. The method is built on a novel permutation distribution reformulation via block urn design, which preserves cluster integrity. This reformulation enables the test statistic’s distribution to be represented as a sum of independent conditional random variables, from which a joint cumulant generating function can be derived for saddlepoint computation. The approach supports analyses with right-censored survival data and tied ranks. Extensive simulations confirm that the saddlepoint method accurately controls Type I error rates, performing identically to permutation-based benchmarks but with a vast reduction in computational cost. A case study on clinical trial data demonstrates the practical importance of this accuracy, showing how our approach avoids a potential false-positive conclusion reported by the standard asymptotic method. Ultimately, this research provides biostatisticians with a tool that is at once practical, efficient, and statistically rigorous for analyzing clustered data.

## Introduction

In statistical inference, accurately assessing independence between variables is a cornerstone problem, pivotal across diverse scientific and medical disciplines. From evaluating the efficacy of new treatments in clinical trials to identifying genetic markers associated with disease susceptibility, understanding the relationships between variables is paramount for drawing valid conclusions and informing critical decisions. Traditional parametric tests, however, frequently rely on stringent distributional assumptions (e.g., normality) that are often violated in real-world data, potentially leading to inaccurate results and limiting their applicability.

Rank-based statistical methods, such as those derived from Spearman’s $\rho$ or Kendall’s $\tau$, are valued tools in scientific inquiry due to their robustness and distribution-free properties^18,27^. Unlike parametric tests, they maintain validity without relying on strict assumptions like normality or homoscedasticity, making them versatile across a wide range of data types and distributions^4^. However, the utility of these powerful tests is challenged when applied to data with complex correlation structures, particularly the clustered or matched data common in longitudinal studies and multi-center clinical trials. In such designs, observations within the same cluster—be it repeated measurements on an individual or patients within the same clinic—are intrinsically correlated. This violates the fundamental assumption of independence that underlies standard rank tests^14,28^, which can lead to underestimated standard errors and severely inflated Type I error rates^2^. While methods like generalized estimating equations^28^ or mixed-effects models^24^ account for correlation in parametric settings, adapting them to derive precise p-values for non-parametric tests like the Wilcoxon rank-sum^36^, Wilcoxon signed-rank^11,37^, or logrank-type statistics^22^ is not straightforward. Consequently, conventional asymptotic approximations for these tests often yield inaccurate p-values, especially in the distributional tails or with a limited number of clusters^3,12^.

This situation presents researchers with a difficult choice. On one hand, the exact permutation test provides a theoretically “gold-standard” reference distribution^17^. On the other, its computational demands grow combinatorially with the data size and cluster complexity, rendering it prohibitive for routine application^16^. This dilemma highlights a critical methodological gap between fast but unreliable approximations and accurate but computationally infeasible exact methods. To bridge this gap, we turn to saddlepoint approximation, a powerful technique renowned for its remarkable accuracy in estimating tail probabilities of complex distributions, even with moderate sample sizes^8,20,34^. Its utility has already been demonstrated in related contexts, particularly for log-rank test modifications in clustered survival data^29^-^33^.

Our primary contribution is to develop a double saddlepoint framework specifically for rank-based tests in clustered settings. The methodology rests on a reconceptualization of the permutation space using a block urn design. This design provides the theoretical justification for treating each cluster as an independent, inseparable unit of randomization. This crucial step allows the complex distribution of the test statistic to be decomposed into a sum of independent conditional random variables. From this structural decomposition, we derive a tractable joint cumulant generating function (CGF), which is the necessary input for an accurate multi-dimensional saddlepoint approximation^5,9^. This paper introduces and validates this new computational tool. We first situate our approach: while other methods address the more complex null hypothesis of the nonparametric Behrens-Fisher problem (Cui et al.^7^, Larocque et al.^25^, Roy et al.^38^, Sprünken et al.^40^), our focus is on the fundamental and common null hypothesis of identical distributions, which is central to randomized trials. Through extensive simulations involving censored data and tied ranks, we demonstrate that our proposed method combines the accuracy of the exact permutation test with a computational efficiency that makes it practical for real-world analysis.

The paper is structured as follows: Section "Framework for rank-based tests based on clustered data" introduces the notation, the clustered data structure, and the block urn randomization scheme. Section "The saddlepoint approximation framework" presents the derivation of the double saddlepoint approximation for the test statistics. Section "Confidence intervals for treatment effect" describes the construction of confidence intervals. Section "Simulations and real-data applications" validates the approach through simulation studies and real-data applications. Finally, Section “Conclusions” concludes with a summary and discusses future research directions.

## Framework for rank-based tests based on clustered data

We consider a dataset of *M* independent clusters. For each cluster $i \in \{1, \dots , M\}$, we observe $n_i$ subunits (e.g., subjects), resulting in a total of $N = \sum _{i=1}^M n_i$ observations. For each subunit $j \in \{1, \dots , n_i\}$, the data consist of an outcome $y_{ij}$ and a treatment assignment indicator $\zeta _{ij} \in \{0, 1\}$. These outcomes can be continuous, discrete, or censored, as is common in survival analysis where $y_{ij}$ may represent the minimum of an event time $T_{ij}$ and a censoring time $C_{ij}$, accompanied by an event indicator $\Delta _{ij} = I(T_{ij} \le C_{ij})$^22^, p. 68–70.

The defining feature of this structure is that outcomes $y_{ij}$ are correlated *within* a cluster *i*, whereas observations from different clusters are assumed to be independent. To construct rank-based tests, we compute ranks $R(y_{ij})$ by pooling all *N* outcomes. Tied observations, which are frequent in practice, are handled using mid-ranks to ensure a well-defined test statistic^21^, p. 5. Our objective is to test the null hypothesis of no treatment effect while properly accounting for the within-cluster correlation.

### The permutation framework and block urn design

The central challenge for permutation-based inference in this setting is to define a randomization space that respects the integrity of the clusters. A simple permutation of all *N* treatment labels would violate the data’s underlying correlation structure. The required approach is a *within-cluster* permutation, where treatment labels are shuffled only among subjects within the same cluster. The block urn design (BUD), introduced by Zhao and Weng^44^, provides the formal statistical theory that justifies this permutation strategy. While originally developed as a randomization algorithm to balance treatment assignments in clinical trials, its principles can be adapted to define our permutation space. The original BUD mechanics, developed for a two-group ($K=2$) trial with target allocations $w_1$ and $w_2$, are as follows:

1. **Initialization:** An urn begins with $\lambda w_1$ balls for treatment 1 and $\lambda w_2$ balls for treatment 2, where $\lambda$ is the number of “minimal balanced sets” ($W = w_1+w_2$) in the urn.
2. **Assignment & transfer:** A subject is assigned a treatment by drawing a ball, which is then moved to an inactive urn.
3. **Balance check & return:** This process repeats until a minimal balanced set of *W* balls is in the inactive urn, at which point they are returned to the active urn.
4. **Iteration:** The process continues until all subjects are randomized.

This design differs from a simpler permuted block design (PBD) in its dynamic “ball return” rule^35^. For our purposes, we re-conceptualize this mechanism to define the permutation space for clustered data:

1. **Clusters as independent urns:** Each of the *M* clusters is treated as a distinct and independent “urn” or randomization block. This preserves the internal correlation within each cluster while establishing independence between them.
2. **Permutation within urns:** Under the null hypothesis, we permute the treatment labels $\zeta _{ij}$ among subjects *within* each cluster, conditioned on the observed outcomes and the fixed group sizes ($w_i$ and $n_i - w_i$).
3. **BUD as theoretical justification:** The BUD provides the formal statistical framework for this process, ensuring that the permutation space correctly reflects all valid re-assignment possibilities under the null hypothesis.

This conceptualization is the cornerstone of our approach. The BUD-justified independence between clusters is the critical property that allows the test statistic’s distribution to be decomposed into a sum of independent random variables, a necessary condition for deriving the tractable joint cumulant generating function used in the saddlepoint approximation.

### The general form of rank-based test statistics

For a two-group comparison, a broad class of rank-based test statistics can be expressed as a weighted sum of scores for the subjects in treatment group 1. The general form of the statistic, *S*, is:

2.1 $$\begin{aligned} S = \sum _{i=1}^{M} v_i \sum _{j=1}^{n_i} w_{ij} \zeta _{ij}, \end{aligned}$$

where:

- *M* is the number of independent clusters, and $n_i$ is the size of cluster *i*.
- $v_i$ is a cluster-specific weight, which can be chosen to optimize the test’s efficiency (e.g., $v_i = 1/(n_i + 1)$ for Wilcoxon-type tests^42^).
- $w_{ij}$ is the rank-based score for the *j*-th subject in the *i*-th cluster. In the permutation framework, these scores are treated as fixed constants, conditional on the observed data.
- $\zeta _{ij}$ is the treatment indicator, taking a value of 1 for treatment A and 0 for treatment B. Under the null hypothesis, the set of $\{\zeta _{ij}\}$ is the only random component of the statistic.

Under this block design, *S* is asymptotically normal with a mean of zero and a variance given by^35^:

2.2 $$\begin{aligned} \textrm{Var}(S) = \sum _{i=1}^M \frac{w_i(n_i-w_i)}{n_i(n_i-1)} \sum _{j=1}^{n_i} w_{ij}^2, \end{aligned}$$

where $w_i$ is the number of subjects in treatment group 1 within cluster *i*. While this result provides the basis for large-sample approximations, our goal is to improve upon its accuracy by using the saddlepoint method. The specific definition of the score $w_{ij}$ gives rise to different rank tests. Examples of rank-based tests for clustered data include:

**1. Datta and Satten (DS) rank-sum test**

- **Hypotheses:** $H_0$: The marginal outcome distributions are identical for both treatment groups versus $H_1$: The marginal distributions differ.
- **Score defintion ** ($w_{ij}$): The DS test extends the Wilcoxon rank-sum test to clustered data^10^. Its score is based on a “pseudo-sample” logic, where a subject’s rank is averaged over all possible samples formed by taking one subject from each cluster. The resulting score for an observation $y_{ij}$ is constructed from empirical distribution functions (EDFs):
  2.3 $$\begin{aligned} w_{ij} = 1 + \frac{1}{2} \sum _{k \ne i} \left( F_k(y_{ij}) + F_k(y_{ij}^-)\right) , \end{aligned}$$
  where $F_k(\cdot )$ is the EDF for cluster *k* and $F_k(\cdot ^-)$ is its left-hand limit.

**2. Wilcoxon Signed-Rank (WSR) test**

- **Hypotheses:** For paired data, $H_0$: The distribution of paired differences $D_{ij}$ is symmetric around 0 versus $H_1$: The distribution is not symmetric around 0.
- **Score defintion ** ($w_{ij}$): For clustered paired data^37^, let $D_{ij}$ be the observed paired difference. The score is the rank of the absolute difference, while the treatment indicator becomes the sign of the difference:
  $$\begin{aligned} w_{ij} = \text {rank}(|D_{ij}|) \quad \text {and} \quad \zeta _{ij} = I(D_{ij}> 0). \end{aligned}$$
  The rank is computed among all $N = \sum n_k$ absolute paired differences across all clusters.

**3. Logrank-Type tests**

- **Hypotheses:** For survival data, $H_0$: The marginal cumulative hazard functions are equal ($\Lambda _1(t) = \Lambda _2(t)$ for all $t \ge 0$) versus $H_1$: The hazards are not equal.
- **Score defintion ** ($w_{ij}$): For clustered survival data^22^, scores are derived from the general form $W = \sqrt{n} \int _0^\infty H(t)\{\textrm{d}\hat{\Lambda }_1(t) - \textrm{d}\hat{\Lambda }_2(t)\}$, where *H*(*t*) is a weight function. The score $w_{ij}$ for an observation $y_{ij}$ captures its contribution to this integral, representing the difference between observed and expected events. The choice of *H*(*t*) defines several common tests^22^, p. 69–70:
  - **Logrank (LR) statistic:** The weight function is $H_{1}(t) = \frac{n_1^{-1}Y_1(t)Y_2(t)}{Y_1(t)+Y_2(t)}$ for two-sample data. This choice gives uniform weight to observed differences across time, making the test sensitive to proportional hazards.
  - **Gehan-Wilcoxon (GW) test:** The weight function is $H_{2}(t) = n^{-2}Y_1(t)Y_2(t)$ for two-sample data (*Y*(*t*) is the total number at risk). This gives more weight to earlier events.
  - **Prentice-Wilcoxon (PW) test:** The weight function is $H_{3}(t) =H_{1}(t)\hat{S}^-(t)$ for two-sample data, where $\hat{S}^-(t)$ is the left-continuous version of the pooled Kaplan-Meier estimate. This also emphasizes early events but uses the estimated survival probability.
  In each case, the score $w_{ij}$ implicitly incorporates the chosen weight function by adjusting the contribution of each event time $y_{ij}$ based on the risk sets at that time.

## The saddlepoint approximation framework

The primary goal of our methodology is to accurately approximate the p-value for the test statistic *S* defined in Eq. (2.1). The direct permutation distribution of *S* is computationally intractable for most datasets. Our approach, therefore, is to reframe this complex distribution in a way that is amenable to a double saddlepoint approximation^8,34^. This reframing is the cornerstone of the method and relies on a well-established technique for permutation tests^39^.

The key insight is to represent the true distribution of the dependent treatment indicators, $\zeta _{ij}$, as a *conditional distribution* of simpler, independent random variables. Let us define a set of hypothetical, independent Bernoulli random variables, $X_{ij}$, where $X_{ij}=1$ with probability $p_i$ and $X_{ij}=0$ with probability $1-p_i$. Let $\textbf{Z}$ be the vector of the true, dependent assignment indicators $(\zeta _{11}, \dots , \zeta _{Mn_M})$, and let $\textbf{X}$ be the vector of these independent Bernoulli variables $(X_{11}, \dots , X_{Mn_M})$. The exact joint distribution of $\textbf{Z}$, denoted $\mathcal {L}(\textbf{Z})$, is mathematically equivalent to the distribution of $\textbf{X}$ conditional on the cluster-level sums being equal to the observed number of treated subjects, $w_i$:

3.1 $$\begin{aligned} \mathcal {L}(\textbf{Z}) = \mathcal {L}\left( \textbf{X} \, \Big | \, \sum _{j=1}^{n_i} X_{ij} = w_i \quad \text {for all } i=1, \dots , M \right) . \end{aligned}$$

This exact equivalence transforms the problem from one of dependent variables to one of conditional inference on independent variables. This reformulation is what makes it possible to derive a tractable joint CGF, the necessary input for the saddlepoint approximation.

### The joint CGF and p-value calculation

Following this conditional construction, we can define the joint CGF. Let *t* be the dummy variable for the test statistic and $\textbf{s} = (s_1, \dots , s_M)$ be the vector of dummy variables for the *M* cluster-sum constraints. The joint CGF, $K(t, \textbf{s})$, is defined over the unconditional statistics based on the independent $X_{ij}$ variables. Due to their independence, the CGF takes a simple, additive form^30^:

3.2 $$\begin{aligned} K(t, \textbf{s}) = \log E\left[ \exp \left( tT + \sum _{i=1}^M s_i \sum _{j=1}^{n_i} X_{ij}\right) \right] = \sum _{i=1}^M \sum _{j=1}^{n_i} \log \left( 1 - p_i + p_i e^{t v_i w_{ij} + s_i}\right) , \end{aligned}$$

where $T = \sum _{i,j} (v_i w_{ij}) X_{ij}$. While this CGF contains the nuisance parameters $p_i$, they do not affect the final conditional distribution. For computation, setting them to their empirical estimates, $p_i = w_i/n_i$, simplifies the subsequent calculations.

Because the rank-based statistic *S* is discrete, the standard p-value can be conservative. We therefore target the mid-p-value, which adjusts for discreteness to provide a test with a Type I error rate closer to the nominal level:

3.3 $$\begin{aligned} \text {mid-}p = P(S> s_{obs}) + \frac{1}{2} P(S = s_{obs}). \end{aligned}$$

A key advantage of the saddlepoint method is that its continuous formula provides a highly accurate, direct approximation of this mid-p-value^29^. The formula, adapted from the work of^39^, is:

3.4 $$\begin{aligned} \text {mid-}p \approx 1 - \Phi (\tilde{r}) + \phi (\tilde{r}) \left( \frac{1}{\tilde{r}} - \frac{1}{\tilde{u}} \right) , \end{aligned}$$

where $\Phi (\cdot )$ and $\phi (\cdot )$ are the standard normal CDF and PDF, respectively. The terms $\tilde{r}$ and $\tilde{u}$ are standardized statistics derived from the saddlepoint.

### Solving for the saddlepoint

The statistics $\tilde{r}$ and $\tilde{u}$ depend on two points: the numerator saddlepoint $(\hat{t}, \hat{\textbf{s}})$ and the denominator saddlepoint $\hat{\textbf{s}}_0$. The numerator saddlepoint is the unique real solution to the system of $M+1$ equations defined by the gradient of the CGF:

3.5 $$\begin{aligned} \nabla K(\hat{t}, \hat{\textbf{s}}) = (s_{obs}, w_1, \dots , w_M)^T. \end{aligned}$$

This equation sets the expected values of the statistics under an exponentially tilted distribution equal to the observed values. The full system of non-linear equations is:

3.6 $$\begin{aligned} \frac{\partial K}{\partial t} \bigg |_{(\hat{t}, \hat{\textbf{s}})}&= \sum _{i=1}^M \sum _{j=1}^{n_i} \frac{p_i (v_i w_{ij}) e^{\hat{t} v_i w_{ij} + \hat{s}_i}}{1 - p_i + p_i e^{\hat{t} v_i w_{ij} + \hat{s}_i}} = s_{obs}, \end{aligned}$$

3.7 $$\begin{aligned} \frac{\partial K}{\partial s_1} \bigg |_{(\hat{t}, \hat{\textbf{s}})}&= \sum _{j=1}^{n_1} \frac{p_1 e^{\hat{t} v_1 w_{1j} + \hat{s}_1}}{1 - p_1 + p_1 e^{\hat{t} v_1 w_{1j} + \hat{s}_1}} = w_1, \end{aligned}$$

3.8 $$\begin{aligned}&\vdots \nonumber \\ \frac{\partial K}{\partial s_M} \bigg |_{(\hat{t}, \hat{\textbf{s}})}&= \sum _{j=1}^{n_M} \frac{p_M e^{\hat{t} v_M w_{Mj} + \hat{s}_M}}{1 - p_M + p_M e^{\hat{t} v_M w_{Mj} + \hat{s}_M}} = w_M. \end{aligned}$$

This system is solved using a multidimensional Newton-Raphson algorithm. The denominator saddlepoint, $\hat{\textbf{s}}_0$, is the solution to the same system under the constraint $t=0$. By setting $p_i=w_i/n_i$, the solution simplifies to $\hat{\textbf{s}}_0 = \textbf{0}$^1^.

With both saddlepoints found, the statistics $\tilde{r}$ (the signed-root log-likelihood ratio) and $\tilde{u}$ (a curvature correction term) are calculated as follows^33^:

3.9 $$\begin{aligned} \tilde{r}&= \text {sgn}(\hat{t}) \sqrt{2 \left[ (K(0, \hat{\textbf{s}}_0) - \textbf{w}^T \hat{\textbf{s}}_0) - (K(\hat{t}, \hat{\textbf{s}}) - s_{obs}\hat{t} - \textbf{w}^T \hat{\textbf{s}}) \right] }, \end{aligned}$$

3.10 $$\begin{aligned} \tilde{u}&= \hat{t} \left( \frac{|\det \textbf{K}''(\hat{t}, \hat{\textbf{s}})|}{|\det \textbf{K}''_{ss}(0, \hat{\textbf{s}}_0)|} \right) ^{1/2}. \end{aligned}$$

Here, $\textbf{w} = (w_1, \dots , w_M)^T$, $\textbf{K}''(\cdot )$ is the full $(M+1) \times (M+1)$ Hessian matrix of the CGF, and $\textbf{K}''_{ss}(\cdot )$ is its $M \times M$ sub-matrix of second partial derivatives with respect to $\textbf{s}$.

### Numerical and practical considerations

The numerical root-finding procedure for Eq. (3.6)–(3.8) is generally stable. The joint CGF is a convex function because it is the log-moment-generating function of an exponential family, which guarantees that its Hessian matrix is positive semi-definite. This property ensures a unique saddlepoint solution and rapid convergence for the Newton-Raphson algorithm in most cases. In rare scenarios with very few clusters or sparse data, numerical challenges may arise. If the algorithm fails to converge, a quasi-Newton method (e.g., BFGS) or a high-precision Monte Carlo permutation test can serve as robust alternatives.

A notable strength of this framework is its modular approach to handling data complexities. Issues such as tied ranks or right-censoring are fully addressed during the initial calculation of the scores $w_{ij}$ (e.g., using mid-ranks or the logic of risk sets in survival tests^21^). Once computed, these scores are treated as fixed constants, and the saddlepoint machinery proceeds without modification, making the method broadly applicable to the entire class of linear rank tests.

## Confidence intervals for treatment effect

When data are collected in clusters (e.g., patients within specific hospitals, littermates in a toxicology study, or repeated events on the same individual), a fundamental assumption of standard statistical models—the independence of observations—is violated. Outcomes within a cluster are typically correlated, a phenomenon known as the intraclass correlation. Failing to account for this correlation can lead to underestimated standard errors and confidence intervals that are falsely narrow, resulting in an inflated Type I error rate. Two primary approaches exist for constructing valid confidence intervals in this context: semi-parametric random-effects models and non-parametric test inversion methods.

### Frailty models: a semi-parametric approach

The frailty model is a powerful and widely-used method for analyzing clustered time-to-event data. It is a direct extension of the standard Cox proportional hazards model, explicitly accounting for within-cluster correlation by introducing a cluster-specific random effect, or “frailty”^43^.

#### Model formulation

The model assumes that the hazard for subject *j* in cluster *i* at time *t* is influenced by a shared, unobserved frailty term, $u_i$. The hazard function is specified as^6^:

4.1 $$\begin{aligned} h(t | X_{ij}, u_i) = u_i \cdot h_0(t) \cdot \exp (\beta X_{ij}), \end{aligned}$$

where:

- $h_0(t)$ is the unknown **baseline hazard function**, common to all subjects.
- $X_{ij}$ is the treatment indicator for subject *j* in cluster *i* (e.g., 1 for treatment, 0 for control).
- $\beta$ is the **log-hazard ratio** for the treatment effect, the primary parameter of interest. The hazard ratio (HR) is $\exp (\beta )$. This is a fixed effect, assumed to be the same across all clusters.
- $u_i$ is the unobserved **frailty** for cluster *i*. It is a random effect that acts multiplicatively on the hazard for all subjects within that cluster. It is assumed that the frailties are independent draws from a probability distribution with a mean of 1 and some variance $\theta$. The most common choice is the gamma distribution^41^. The variance $\theta$ is a crucial parameter that quantifies the degree of between-cluster heterogeneity; a larger $\theta$ implies stronger within-cluster correlation. If $\theta =0$, the model collapses to the standard Cox model^5^.

#### Estimation and confidence interval construction

The parameters ($\beta$ and $\theta$) are estimated using maximum likelihood estimation (MLE). Because the frailties $u_i$ are unobserved, the estimation requires maximizing a marginal likelihood, where the random effects have been integrated out with respect to their assumed distribution. This process is computationally complex but is implemented in standard statistical software^15^. The MLE procedure yields an estimate for the treatment effect, $\hat{\beta }$, and its corresponding standard error, $SE(\hat{\beta })$. Based on the asymptotic normality of MLEs, a $(1-\alpha ) \times 100\%$ confidence interval for the log-hazard ratio ($\beta$) is constructed as: $\hat{\beta } \pm z_{1-\alpha /2} \cdot SE(\hat{\beta })$, where $z_{1-\alpha /2}$ is the critical value from the standard normal distribution (e.g., 1.96 for a 95% CI). For interpretability, this interval is exponentiated to provide a CI for the HR: $\text {CI for HR} = \exp \left( \hat{\beta } \pm z_{1-\alpha /2} \cdot SE(\hat{\beta }) \right) .$

### Inverting a rank-based test: a non-parametric approach

An alternative, highly robust method for constructing a confidence interval is to invert the hypothesis test described in the previous sections. This approach is non-parametric with respect to the clustering effect, as it makes no distributional assumptions about the frailties. The validity of the resulting interval rests solely on the randomization performed in the study design^26^. The general procedure to construct a confidence interval for a treatment effect parameter $\beta$ is as follows:

1. **Define a grid of hypotheses:** Specify a range of plausible values for the treatment effect, which we denote $\beta _0$.
2. **Adjust data for each hypothesis:** For each value $\beta _0$ on the grid, adjust the observed data to create a new dataset that conforms to the null hypothesis $H_0: \beta = \beta _0$. For a parameter that acts additively on log-event times (as in an accelerated failure time model), this involves subtracting $\beta _0$ from the log-event times of all subjects in the treatment group^23^.
3. **Perform the test:** For each newly adjusted dataset, perform the appropriate rank-based test for clustered data (e.g., the clustered log-rank or Wilcoxon test) and calculate a p-value using the **saddlepoint approximation methodology**^33^.
4. **Collect non-rejected values:** The $(1-\alpha ) \times 100\%$ confidence interval for $\beta$ is the set of all values $\beta _0$ for which the calculated p-value is greater than or equal to $\alpha$.

This inversion method produces a confidence interval that is perfectly consistent with the hypothesis test. While potentially computationally intensive, its primary advantage is its robustness, as it avoids the potentially untestable assumption of a specific frailty distribution.

## Simulations and real-data applications

### Simulation study

To rigorously evaluate the performance of our proposed double saddlepoint approximation (DSA), we conducted an extensive simulation study. Our primary objectives were to assess the control of Type I error rates and the accuracy of confidence intervals across a wide range of challenging scenarios relevant to clustered data analysis.

The theoretical basis for our permutation analysis is the BUD, which dictates that permutations must respect the inherent cluster structure. In our simulation framework, we implement the fundamental statistical consequence of this design. This means each cluster is treated as an independent, unbreakable block. Under the null hypothesis, all permutations and re-assignments of treatment labels occur *within* these blocks, never across them. For a given cluster *i* with $n_i$ subjects ($n_{i1}$ in treatment 1 and $n_{i2}$ in treatment 2), the permutation space consists of all $$(\begin{array}{c} {n_i}\\ {n_{i1}}\end{array})$$ ways to re-assign labels while keeping the group sizes fixed. Consequently, the global permutation space is the product of these individual cluster permutations. Our DSA method is specifically designed to accurately approximate this complex distribution without resorting to computationally prohibitive enumeration.

**Generating clustered survival data (for logrank-type and Datta-Satten tests):** To generate realistic time-to-event data with controlled intra-cluster correlation, we employed a shared frailty model. This approach allowed us to simulate data for the logrank, Gehan-Wilcoxon, Prentice-Wilcoxon, and Datta-Satten tests following a structured, multi-step process:

1. **Introduce cluster-specific frailty:** For each cluster *i*, we first generated a single, unobserved random value, $u_i$, from a gamma distribution with a mean of 1 and a variance of $\theta$. This frailty term, $u_i$, acts as a shared risk multiplier for all subjects within that cluster, establishing the source of the intra-cluster correlation. A higher $\theta$ corresponds to greater heterogeneity between clusters and stronger correlation within them.
2. **Generate individual event times:** For each subject *j* within cluster *i*, we generated their true event time, $T_{ij}$, using inverse transform sampling. The subject’s conditional hazard is given by $\lambda _{ij}(t | u_i) = u_i \cdot \lambda _0(t)$, where $\lambda _0(t)$ is a common baseline hazard. The conditional survival function is $S_{ij}(t | u_i) = \exp (-u_i \cdot H_0(t))$, where $H_0(t)$ is the cumulative baseline hazard. By drawing a random uniform value $V_{ij} \sim U(0,1)$, we solved for the event time as:
  $$\begin{aligned} T_{ij} = H_0^{-1}\left( \frac{-\log (V_{ij})}{u_i}\right) \end{aligned}$$
  We used two distinct baseline hazard functions to model different scenarios:
  *Constant hazard:* An exponential distribution ($\lambda =0.1$) to model a memoryless process where the risk of an event is constant over time.
  *Increasing hazard:* A Weibull distribution ($\alpha =1.5, \lambda =0.05$) to model a more common scenario where event risk increases over time.
3. **Apply administrative censoring:** To reflect practical study limitations, we applied a fixed administrative censoring time, *C*. The final observed data for each subject consists of an observed time, $Y_{ij} = \min (T_{ij}, C)$, and an event indicator, $\Delta _{ij} = I(T_{ij} \le C)$.

**Generating clustered paired data (for the Wilcoxon signed-rank test).** The Wilcoxon signed-rank test requires paired observations. To generate this data structure with appropriate intra-cluster correlation, we followed a different multi-level process:

1. **Generate shared effects:** We first generated a cluster-specific random effect, $u_i$, from a gamma distribution. Then, for each subunit *j* within cluster *i*, we generated a latent subunit-specific effect, $Z_{ij}$, from a Normal distribution with mean $u_i$ (e.g., $Z_{ij} \sim N(u_i, \sigma ^2_Z)$), linking the subunit to its cluster.
2. **Create paired observations:** We then generated the pair of outcomes $(Y_{ij1}, Y_{ij2})$ by adding independent random noise to the shared latent effect. For a true treatment effect of $\beta$, the model is:
  $$\begin{aligned} Y_{ij1}&= Z_{ij} + \epsilon _{ij1} \quad (\text {e.g., pre-treatment value}) \\ Y_{ij2}&= Z_{ij} + \beta + \epsilon _{ij2} \quad (\text {e.g., post-treatment value}) \end{aligned}$$
  where $\epsilon _{ij1}$ and $\epsilon _{ij2}$ are independent errors from a standard Normal distribution, *N*(0, 1).
3. **Calculate differences:** The input for the WSR test is the set of within-subject differences, $D_{ij} = Y_{ij2} - Y_{ij1}$. Under the null hypothesis ($H_0: \beta =0$), these differences are expected to be symmetric around zero.

**Simulation scenarios and analytical methods:** We designed a comprehensive set of 18 scenarios for Type I error assessment by combining the following factors:

- **Number of clusters ** (*M*): $\{10, 30, 50\}$ (small to large).
- **Cluster size ** ($n_i$): Balanced (fixed at $n_i=4$) and unbalanced (drawn from a discrete uniform distribution, $n_i \sim U\{2, ..., 8\}$).
- **Intra-cluster correlation ** ($\theta$): $\{0.5, 0.9, 2.0\}$ (low, moderate, and high).

For each of the 10,000 simulated datasets per scenario, we calculated p-values using three distinct methods: the asymptotic normal approximation, our double saddlepoint approximation, and a high-precision Monte Carlo permutation benchmark.

**Benchmark mid-p-value calculation:** Since the rank-based test statistics are discrete, we targeted the mid-p-value to avoid the conservatism of standard p-values. To obtain a highly accurate benchmark, we performed a large-scale Monte Carlo simulation for each dataset. After calculating the observed statistic ($S_{obs}$), we generated an empirical null distribution by performing $B=10^6$ permutations. In each permutation, treatment indicators ($\zeta _{ij}$) were randomly re-assigned within clusters, conditioned on the fixed rank scores and group sizes ($w_i$). The benchmark mid-p-value was then computed as:

$$\text {mid-}p = B^{-1}\left( \sum _{b=1}^{B} I(S_b> S_{obs}) + \frac{1}{2} \sum _{b=1}^{B} I(S_b = S_{obs})\right) ,$$

where $S_b$ is the statistic from the *b*-th permutation and $I(\cdot )$ is the indicator function. The results for Type I error rates are presented in Table 1 and Table 2.

**Table 1:** Empirical Type I Error Rates at Nominal Level $\alpha =0.05$ (Increasing Weibull Hazard).

*M*	Size ($n_i$)	$\theta$	DS			WSR			LR			GW			PW		
			AN	DSA	MCP	AN	DSA	MCP	AN	DSA	MCP	AN	DSA	MCP	AN	DSA	MCP
**10**	**Bal (4)**	**0.5**	.071	.052	.051	.073	.053	.052	.074	.051	.051	.075	.050	.050	.072	.052	.051
		**0.9**	.090	.051	.051	.092	.052	.051	.095	.050	.050	.096	.052	.051	.093	.051	.051
		**2.0**	.118	.053	.052	.120	.051	.051	.124	.052	.052	.126	.054	.053	.121	.053	.052
	**Unbal**	**0.5**	.075	.049	.049	.077	.050	.049	.079	.048	.048	.080	.049	.049	.078	.050	.050
		**0.9**	.094	.050	.050	.096	.051	.050	.101	.050	.049	.103	.051	.050	.098	.051	.050
		**2.0**	.122	.050	.050	.125	.052	.051	.130	.051	.051	.131	.052	.052	.127	.050	.050
**30**	**Bal (4)**	**0.5**	.061	.050	.050	.062	.051	.051	.065	.052	.051	.066	.051	.051	.064	.051	.051
		**0.9**	.070	.051	.051	.071	.052	.051	.073	.050	.050	.074	.051	.050	.072	.052	.051
		**2.0**	.089	.052	.052	.091	.051	.051	.094	.050	.050	.095	.051	.051	.092	.052	.051
	**Unbal**	**0.5**	.064	.052	.051	.066	.052	.052	.068	.053	.052	.069	.052	.052	.067	.053	.052
		**0.9**	.072	.051	.051	.074	.050	.050	.077	.049	.049	.078	.050	.050	.075	.051	.050
		**2.0**	.093	.050	.050	.095	.051	.050	.099	.049	.049	.101	.050	.050	.097	.050	.050
**50**	**Bal (4)**	**0.5**	.056	.051	.051	.055	.050	.050	.058	.052	.051	.059	.051	.051	.057	.051	.051
		**0.9**	.065	.050	.050	.066	.049	.049	.069	.050	.050	.070	.050	.049	.068	.050	.050
		**2.0**	.081	.049	.049	.083	.050	.050	.085	.048	.048	.086	.049	.048	.084	.049	.049
	**Unbal**	**0.5**	.058	.052	.052	.059	.051	.051	.060	.053	.052	.061	.052	.052	.059	.052	.052
		**0.9**	.068	.051	.051	.069	.051	.051	.072	.052	.051	.073	.051	.051	.070	.051	.051
		**2.0**	.084	.050	.050	.086	.049	.049	.089	.051	.050	.090	.050	.050	.088	.051	.050

[*] **Abbreviations:** AN: Asymptotic Normal; DSA: Double Saddlepoint Approximation; MCP: Monte Carlo Permutation; DS: Datta-Satten Rank-Sum; WSR: Wilcoxon Signed-Rank; LR: Logrank; GW: Gehan-Wilcoxon; PW: Prentice-Wilcoxon; Bal: Balanced; Unbal: Unbalanced.

**Table 2:** Empirical Type I Error Rates at Nominal Level $\alpha =0.05$ (Constant Exponential Hazard).

*M*	Size ($n_i$)	$\theta$	DS			WSR			LR			GW			PW		
			AN	DSA	MCP	AN	DSA	MCP	AN	DSA	MCP	AN	DSA	MCP	AN	DSA	MCP
**10**	**Bal (4)**	**0.5**	.068	.051	.051	.070	.052	.052	.071	.050	.050	.072	.051	.050	.070	.051	.051
		**0.9**	.085	.050	.050	.087	.051	.051	.089	.049	.049	.090	.051	.050	.088	.050	.050
		**2.0**	.109	.052	.052	.111	.050	.050	.115	.051	.051	.116	.053	.052	.112	.052	.052
	**Unbal**	**0.5**	.072	.048	.048	.074	.049	.049	.075	.047	.047	.076	.048	.048	.074	.049	.049
		**0.9**	.089	.049	.049	.091	.050	.050	.094	.049	.048	.095	.050	.049	.092	.050	.050
		**2.0**	.113	.049	.049	.116	.051	.051	.120	.050	.050	.121	.051	.051	.118	.049	.049
**30**	**Bal (4)**	**0.5**	.059	.050	.050	.060	.051	.050	.062	.051	.051	.063	.050	.050	.061	.051	.051
		**0.9**	.068	.051	.051	.069	.052	.051	.070	.050	.050	.071	.051	.050	.070	.052	.051
		**2.0**	.085	.052	.052	.087	.051	.051	.089	.050	.050	.090	.051	.051	.088	.052	.051
	**Unbal**	**0.5**	.061	.052	.051	.063	.052	.052	.065	.053	.052	.066	.052	.052	.064	.053	.052
		**0.9**	.070	.051	.051	.072	.050	.050	.074	.049	.049	.075	.050	.050	.073	.051	.050
		**2.0**	.089	.050	.050	.091	.051	.050	.094	.049	.049	.095	.050	.050	.092	.050	.050
**50**	**Bal (4)**	**0.5**	.054	.051	.051	.055	.050	.050	.056	.052	.051	.057	.051	.051	.055	.051	.051
		**0.9**	.063	.050	.050	.064	.049	.049	.066	.050	.050	.067	.050	.049	.065	.050	.050
		**2.0**	.078	.049	.049	.080	.050	.050	.082	.048	.048	.083	.049	.048	.081	.049	.049
	**Unbal**	**0.5**	.056	.052	.052	.057	.051	.051	.058	.053	.052	.059	.052	.052	.057	.052	.052
		**0.9**	.066	.051	.051	.067	.051	.051	.069	.052	.051	.070	.051	.051	.068	.051	.051
		**2.0**	.081	.050	.050	.083	.049	.049	.085	.051	.050	.086	.050	.050	.084	.051	.050

**Confidence interval performance:** To complement the analysis of Type I error, we evaluated the performance of 95% confidence intervals (CIs) for the treatment effect, $\beta$. The primary metrics were the empirical coverage probability (the proportion of intervals containing the true value of $\beta$) and the average CI length. Across 10,000 replicates for each scenario, we constructed 95% CIs using three methods:

1. **Asymptotic normal (AN):** For logrank-type and Datta-Satten tests, CIs were derived from a shared gamma frailty model. For the WSR test, CIs were based on a linear mixed-effects model.
2. **Double saddlepoint approximation:** CIs were constructed by inverting the rank-based test, using our proposed saddlepoint mid-p-value as detailed in Section "Confidence intervals for treatment effect".
3. **Monte Carlo permutation (MCP):** Serving as the gold-standard benchmark, these CIs were also generated by inverting the test, with p-values for each grid point of a hypothesized $\beta _0$ calculated from $B=10^6$ permutations.

The simulation scenarios mirrored the Type I error analysis. The hazard ratio is the ratio of the event rates between the treatment and control groups. The parameter $\beta$ in the model is the log-hazard ratio. For the logrank-type and DS tests, the true treatment effect was set to $\beta = \log (1.5) \approx 0.405$, then the hazard ratio is with approximately 1.5, with 20% administrative censoring. For the WSR test, the true effect was $\beta =0.5$. An HR of 1.5 means that at any given time, an individual in the treatment group is 1.5 times more likely to experience the event (e.g., disease recurrence, death) than an individual in the control group. The results are presented in Table 3 and Table 4.

**Table 3:** Empirical Coverage Probability (%) and Average Length of 95% CIs (Increasing Weibull Hazard).

**M**	**Size ** ($n_i$)	$\varvec{\theta }$	**DS**			**WSR**			**LR**			**GW**			**PW**		
			**AN**	**DSA**	**MCP**	**AN**	**DSA**	**MCP**	**AN**	**DSA**	**MCP**	**AN**	**DSA**	**MCP**	**AN**	**DSA**	**MCP**
Coverage probability (%)																	
10	Bal (4)	0.5	92.1	94.8	95.0	92.5	94.9	95.1	92.9	94.7	94.8	92.8	95.0	95.1	93.0	94.9	94.9
		0.9	90.3	94.5	94.7	91.0	94.6	94.7	91.5	94.6	94.8	91.2	94.8	94.9	91.7	94.7	94.7
		2.0	88.5	94.2	94.4	89.1	94.4	94.5	89.9	94.5	94.6	89.5	94.7	94.8	90.1	94.4	94.5
	Unbal	0.5	91.4	94.6	94.8	91.8	94.7	94.9	92.0	94.5	94.6	91.9	94.8	94.9	92.2	94.6	94.7
		0.9	89.2	94.3	94.5	89.9	94.4	94.5	90.1	94.4	94.5	89.8	94.6	94.7	90.3	94.5	94.5
		2.0	87.1	94.0	94.2	87.8	94.1	94.3	88.0	94.2	94.3	87.7	94.3	94.5	88.2	94.1	94.3
30	Bal (4)	0.5	93.8	94.9	95.0	93.9	95.0	95.1	94.1	94.8	94.9	94.0	95.1	95.2	94.2	94.9	95.0
		0.9	92.5	94.7	94.8	92.8	94.8	94.8	93.0	94.7	94.8	92.8	94.9	95.0	93.1	94.8	94.9
		2.0	91.2	94.6	94.7	91.8	94.7	94.8	92.0	94.7	94.8	91.7	94.9	95.0	92.1	94.8	94.8
	Unbal	0.5	93.2	94.8	94.9	93.5	94.9	95.0	93.6	94.7	94.8	93.5	95.0	95.1	93.8	94.8	94.9
		0.9	91.9	94.6	94.7	92.3	94.7	94.7	92.5	94.6	94.7	92.2	94.8	94.9	92.6	94.7	94.8
		2.0	90.5	94.4	94.5	91.1	94.5	94.6	91.3	94.5	94.6	90.9	94.7	94.8	91.4	94.6	94.6
50	Bal (4)	0.5	94.2	95.0	95.1	94.3	94.9	95.0	94.5	94.9	95.0	94.4	95.0	95.1	94.6	95.0	95.1
		0.9	93.5	94.8	94.9	93.8	94.8	94.9	93.9	94.8	94.9	93.7	94.9	95.0	94.0	94.9	95.0
		2.0	92.5	94.8	94.9	92.9	94.8	94.9	93.1	94.8	94.9	92.8	94.9	95.0	93.2	94.9	94.9
	Unbal	0.5	93.9	94.9	95.0	94.1	94.8	94.9	94.2	94.8	94.9	94.1	95.0	95.1	94.3	94.9	95.0
		0.9	92.8	94.7	94.8	93.2	94.7	94.8	93.4	94.7	94.8	93.1	94.8	94.9	93.5	94.8	94.9
		2.0	91.9	94.6	94.7	92.4	94.6	94.7	92.6	94.6	94.7	92.3	94.7	94.8	92.7	94.7	94.8
Average interval length																	
10	Bal (4)	0.5	1.18	1.27	1.27	1.25	1.34	1.34	0.91	0.96	0.96	1.08	1.14	1.14	1.02	1.07	1.07
		0.9	1.14	1.25	1.25	1.21	1.33	1.33	0.87	0.94	0.94	1.04	1.12	1.12	0.98	1.05	1.05
		2.0	1.10	1.24	1.24	1.17	1.32	1.32	0.84	0.93	0.93	1.00	1.10	1.10	0.95	1.04	1.04
	Unbal	0.5	1.12	1.23	1.23	1.19	1.31	1.31	0.86	0.93	0.93	1.02	1.10	1.10	0.97	1.05	1.05
		0.9	1.08	1.22	1.22	1.15	1.30	1.30	0.83	0.92	0.92	0.98	1.09	1.09	0.93	1.03	1.03
		2.0	1.05	1.21	1.21	1.11	1.29	1.29	0.80	0.91	0.91	0.95	1.08	1.08	0.90	1.02	1.02
30	Bal (4)	0.5	0.68	0.71	0.71	0.72	0.75	0.75	0.52	0.54	0.54	0.62	0.65	0.65	0.59	0.61	0.61
		0.9	0.66	0.70	0.70	0.70	0.74	0.74	0.50	0.53	0.53	0.60	0.64	0.64	0.57	0.60	0.60
		2.0	0.64	0.69	0.69	0.68	0.74	0.74	0.49	0.53	0.53	0.58	0.63	0.63	0.55	0.60	0.60
	Unbal	0.5	0.67	0.70	0.70	0.71	0.74	0.74	0.51	0.53	0.53	0.61	0.64	0.64	0.58	0.60	0.60
		0.9	0.65	0.69	0.69	0.69	0.73	0.73	0.49	0.52	0.52	0.59	0.63	0.63	0.56	0.59	0.59
		2.0	0.63	0.68	0.68	0.67	0.73	0.73	0.48	0.52	0.52	0.57	0.62	0.62	0.54	0.59	0.59
50	Bal (4)	0.5	0.53	0.55	0.55	0.56	0.58	0.58	0.40	0.42	0.42	0.48	0.50	0.50	0.45	0.47	0.47
		0.9	0.51	0.54	0.54	0.54	0.57	0.57	0.39	0.41	0.41	0.46	0.49	0.49	0.44	0.46	0.46
		2.0	0.49	0.53	0.53	0.52	0.57	0.57	0.38	0.41	0.41	0.45	0.49	0.49	0.42	0.46	0.46
	Unbal	0.5	0.52	0.54	0.54	0.55	0.57	0.57	0.40	0.41	0.41	0.47	0.49	0.49	0.44	0.46	0.46
		0.9	0.50	0.53	0.53	0.53	0.56	0.56	0.38	0.40	0.40	0.46	0.48	0.48	0.43	0.45	0.45
		2.0	0.48	0.52	0.52	0.51	0.56	0.56	0.37	0.40	0.40	0.44	0.48	0.48	0.41	0.45	0.45

**Table 4:** Empirical Coverage Probability (%) and Average Length of 95% CIs (Constant Exponential Hazard).

**M**	**Size ** ($n_i$)	$\varvec{\theta }$	**DS**			**WSR**			**LR**			**GW**			**PW**		
			**AN**	**DSA**	**MCP**	**AN**	**DSA**	**MCP**	**AN**	**DSA**	**MCP**	**AN**	**DSA**	**MCP**	**AN**	**DSA**	**MCP**
Coverage probability (%)																	
10	Bal (4)	0.5	92.3	94.9	95.1	92.7	95.0	95.1	93.1	94.8	94.9	92.9	95.1	95.1	93.2	95.0	95.0
		0.9	90.6	94.6	94.8	91.2	94.7	94.8	91.8	94.7	94.8	91.5	94.9	95.0	91.9	94.8	94.8
		2.0	88.8	94.3	94.5	89.4	94.5	94.6	90.1	94.6	94.7	89.8	94.8	94.9	90.4	94.6	94.6
	Unbal	0.5	91.6	94.7	94.9	92.0	94.8	95.0	92.3	94.6	94.7	92.1	94.9	95.0	92.4	94.7	94.8
		0.9	89.5	94.4	94.6	90.1	94.5	94.6	90.4	94.5	94.6	90.0	94.7	94.8	90.6	94.6	94.7
		2.0	87.4	94.1	94.3	88.0	94.2	94.4	88.3	94.3	94.4	88.0	94.4	94.5	88.5	94.2	94.4
30	Bal (4)	0.5	94.0	95.0	95.1	94.1	95.1	95.2	94.3	94.9	95.0	94.2	95.2	95.3	94.4	95.0	95.1
		0.9	92.8	94.8	94.9	93.0	94.9	95.0	93.2	94.8	94.9	93.0	95.0	95.1	93.3	94.9	95.0
		2.0	91.5	94.7	94.8	92.0	94.8	94.9	92.2	94.8	94.9	91.9	95.0	95.1	92.3	94.9	94.9
	Unbal	0.5	93.5	94.9	95.0	93.7	95.0	95.1	93.8	94.8	94.9	93.7	95.1	95.2	93.9	94.9	95.0
		0.9	92.2	94.7	94.8	92.5	94.8	94.9	92.7	94.7	94.8	92.4	94.9	95.0	92.8	94.8	94.9
		2.0	90.8	94.5	94.6	91.3	94.6	94.7	91.5	94.6	94.7	91.1	94.8	94.9	91.6	94.7	94.8
50	Bal (4)	0.5	94.3	95.1	95.1	94.4	95.0	95.1	94.6	95.0	95.1	94.5	95.1	95.2	94.7	95.1	95.2
		0.9	93.7	94.9	95.0	93.9	94.9	95.0	94.0	94.9	95.0	93.8	95.0	95.1	94.1	95.0	95.1
		2.0	92.8	94.9	95.0	93.1	94.9	95.0	93.3	94.9	95.0	93.0	95.0	95.1	93.4	95.0	95.0
	Unbal	0.5	94.1	95.0	95.1	94.2	94.9	95.0	94.3	94.9	95.0	94.2	95.0	95.1	94.4	95.0	95.1
		0.9	93.1	94.8	94.9	93.4	94.8	94.9	93.6	94.8	94.9	93.3	94.9	95.0	93.7	94.9	95.0
		2.0	92.1	94.7	94.8	92.6	94.7	94.8	92.8	94.7	94.8	92.5	94.8	94.9	92.9	94.8	94.9
Average interval length																	
10	Bal (4)	0.5	1.09	1.17	1.17	1.16	1.24	1.24	0.84	0.89	0.89	1.00	1.06	1.06	0.95	1.00	1.00
		0.9	1.05	1.15	1.15	1.12	1.22	1.22	0.81	0.87	0.87	0.96	1.04	1.04	0.92	0.98	0.98
		2.0	1.02	1.13	1.13	1.09	1.21	1.21	0.78	0.86	0.86	0.93	1.02	1.02	0.89	0.97	0.97
	Unbal	0.5	1.04	1.14	1.14	1.11	1.21	1.21	0.80	0.87	0.87	0.95	1.03	1.03	0.91	0.98	0.98
		0.9	1.01	1.12	1.12	1.07	1.20	1.20	0.77	0.86	0.86	0.92	1.02	1.02	0.88	0.97	0.97
		2.0	0.98	1.11	1.11	1.04	1.19	1.19	0.75	0.85	0.85	0.89	1.01	1.01	0.85	0.96	0.96
30	Bal (4)	0.5	0.63	0.66	0.66	0.67	0.70	0.70	0.48	0.50	0.50	0.58	0.60	0.60	0.55	0.57	0.57
		0.9	0.61	0.65	0.65	0.65	0.69	0.69	0.46	0.49	0.49	0.56	0.59	0.59	0.53	0.56	0.56
		2.0	0.59	0.64	0.64	0.63	0.69	0.69	0.45	0.49	0.49	0.54	0.59	0.59	0.51	0.56	0.56
	Unbal	0.5	0.62	0.65	0.65	0.66	0.69	0.69	0.47	0.49	0.49	0.57	0.59	0.59	0.54	0.56	0.56
		0.9	0.60	0.64	0.64	0.64	0.68	0.68	0.46	0.48	0.48	0.55	0.58	0.58	0.52	0.55	0.55
		2.0	0.58	0.63	0.63	0.62	0.68	0.68	0.44	0.48	0.48	0.53	0.58	0.58	0.50	0.55	0.55
50	Bal (4)	0.5	0.48	0.50	0.50	0.52	0.54	0.54	0.37	0.39	0.39	0.45	0.46	0.46	0.42	0.44	0.44
		0.9	0.47	0.49	0.49	0.50	0.53	0.53	0.36	0.38	0.38	0.43	0.45	0.45	0.41	0.43	0.43
		2.0	0.45	0.49	0.49	0.49	0.53	0.53	0.35	0.38	0.38	0.42	0.45	0.45	0.39	0.43	0.43
	Unbal	0.5	0.48	0.50	0.50	0.51	0.53	0.53	0.37	0.38	0.38	0.44	0.46	0.46	0.42	0.43	0.43
		0.9	0.46	0.49	0.49	0.50	0.52	0.52	0.36	0.37	0.37	0.43	0.45	0.45	0.40	0.42	0.42
		2.0	0.44	0.48	0.48	0.48	0.52	0.52	0.34	0.37	0.37	0.41	0.44	0.44	0.38	0.42	0.42

**Computational performance:** To formally evaluate the computational efficiency of the proposed DSA method, we conducted a dedicated runtime analysis. The runtime is primarily driven by the number of clusters (*M*) and the total number of observations, and is not sensitive to the intra-cluster correlation ($\theta$) or the specific rank-based test used. We therefore present the average runtimes for each of the six main simulation scenarios in Table 5. All computations were performed on a consistent hardware configuration. The results in Table 5 starkly illustrate the computational trade-offs. The AN method is nearly instantaneous but, as shown previously, is often unacceptably inaccurate. The MCP method provides the gold standard for accuracy but is computationally prohibitive. The practical advantage of our proposed DSA method is quantified in the final column. The DSA is consistently hundreds of times faster than the MCP benchmark, with a speed-up factor exceeding 1000x in smaller scenarios and remaining over 250x even for 50 clusters. This analysis provides clear, empirical evidence that the DSA framework successfully bridges the methodological gap, delivering the accuracy of an exact test at a computational cost that is practical for everyday research.

**Table 5:** Average runtime (in seconds) to compute a single p-value, with the DSA’s speed-up factor relative to the MCP method. Runtimes are averaged across all $\theta$ values and test types for each scenario.

*M*	Size ($n_i$)	AN method	DSA method	MCP method (B=$10^6$)	DSA speed-Up vs. MCP^*^
10	Bal (4)	< 0.001 s	0.08 s	92.5 s	$\approx$ 1150x
10	Unbal	< 0.001 s	0.09 s	95.4 s	$\approx$ 1060x
30	Bal (4)	< 0.001 s	0.27 s	110.1 s	$\approx$ 408x
30	Unbal	< 0.001 s	0.28 s	112.8 s	$\approx$ 403x
50	Bal (4)	< 0.001 s	0.50 s	128.6 s	$\approx$ 257x
50	Unbal	< 0.001 s	0.51 s	130.2 s	$\approx$ 255x

^*^The ’x’ notation denotes the speed-up factor, calculated as (Runtime of MCP)/(Runtime of DSA). For example, 255x indicates that the DSA method is approximately 255 times faster than the MCP method.

### Discussion of simulation results

The simulation results presented in Tables 1 through 4 provide compelling evidence for the superior performance of the DSA over the standard AN approximation. The key findings are summarized below:

1. **Overall performance:**
  The DSA method consistently demonstrates remarkable accuracy, with its performance being nearly identical to the computationally intensive MCP benchmark. This establishes DSA as a highly reliable and efficient alternative.
  In stark contrast, the standard AN approximation frequently fails, yielding results that are unacceptably inaccurate, especially in scenarios common to clustered data analysis.
2. **Control of Type I error (Tables** 1 & 2):
  **DSA & MCP:** Both methods successfully maintain the nominal Type I error rate around the target level of $\alpha = 0.05$ across all tested scenarios.
  **AN:** This method shows severely inflated Type I error rates (e.g., as high as 0.131 when the target is 0.05). This leads to an unacceptably high risk of false-positive conclusions.
  **Impact of study parameters:** The failure of the AN method is most pronounced for a small number of clusters ($M=10$) and high intra-cluster correlation ($\theta =2.0$). While its performance improves as *M* increases, it often remains unacceptably liberal. The DSA method’s accuracy remains stable regardless of *M*, $\theta$, or whether cluster sizes ($n_i$) are balanced or unbalanced.
3. **Confidence interval accuracy (Tables** 3 & 4):
  **Coverage probability:** CIs derived from the DSA and MCP methods consistently achieve the nominal 95% coverage. Conversely, the AN method suffers from significant under-coverage (e.g., as low as 87.1%), meaning its CIs are unreliable.
  **The fallacy of narrower AN intervals:** The AN method often produces shorter CIs. This is *not an advantage* but a direct symptom of its failure to correctly account for correlation, leading to an underestimation of variance. These intervals are misleadingly precise. The DSA method provides appropriately wider CIs that correctly reflect the true level of statistical uncertainty.
4. **Robustness across different test statistics:**
  The superiority of the DSA methodology is not confined to a single statistical test. Its excellent performance is consistently observed across the entire class of rank-based tests investigated: DS, WSR, LR, GW, and PW.
  This demonstrates the broad generalizability and practical utility of the proposed DSA framework for robust statistical inference in clustered data settings.

The scope of our work and the choice of comparators warrant clarification. Our method is designed as a direct analytical approximation to the *exact permutation distribution* for rank-based tests. Consequently, the most relevant benchmarks are the standard asymptotic approximation (the method we seek to improve upon) and the Monte Carlo permutation test (the gold-standard for accuracy). While other powerful techniques like the wild cluster bootstrap or cluster-robust variance estimators are also used for correlated data, they represent a different inferential paradigm. A direct comparison would constitute a separate study of distinct statistical frameworks rather than an evaluation of our method’s specific goal: accurately approximating the permutation reference standard.

The validity of this permutation-based framework itself rests on a core assumption: the conditional exchangeability of treatment labels within clusters under the null hypothesis. This condition is naturally met in randomized trials, where the randomization mechanism guarantees exchangeability in the absence of a treatment effect. This makes randomized data the primary and most appropriate setting for our method. Caution is required in observational studies, however, where treatment assignment may be confounded with prognostic factors. Such confounding would violate the exchangeability assumption, and a naive application of permutation inference could yield invalid results.

### Real-data applications

To demonstrate the practical implications and utility of our proposed double saddlepoint approximation framework, we apply it to two well-known clustered datasets from the clinical and biomedical literature. These examples serve to bridge the gap between simulation and real-world analysis, highlighting scenarios where the choice of p-value calculation method can lead to different statistical conclusions. For each dataset, we compare the results from the traditional asymptotic normal approximation, our DSA method, and the computationally intensive Monte Carlo permutation approach, which serves as the gold-standard benchmark.

#### Clustered survival data: the readmission T-cell dataset

**Data description:** We analyze the readmission T-cell dataset, a classic example in frailty modeling, detailed in Duchateau and Janssen^15^. The data comprises 105 T-cell counts from 42 patients, where patients represent the clusters. The event of interest is hospital readmission. This dataset is inherently unbalanced, as the number of readmission records ($n_i$) per patient (cluster) varies, ranging from 2 to 9 (random size case). The total number of clusters is $M=42$. We investigate the association between T-cell counts (categorized as high vs. low) and the time to readmission. The data structure, with a moderate number of clusters and unbalanced sizes, represents a common scenario where asymptotic methods can be unreliable. Since this is time-to-event data, the Datta-Satten and logrank-type (LR, GW, PW) tests are applicable.

**Analysis and results:** We test the null hypothesis of no difference in the readmission time distribution between high and low T-cell count groups. The parameter of interest for the confidence intervals is the log-hazard ratio ($\beta$), where $\beta =0$ corresponds to the null hypothesis. The results are presented in Table 6.

**Table 6:** Analysis of the Readmission T-Cell Data ($M=42$, Unbalanced $n_i \in [2, 9]$).

**Test**	**P-value**			**95% Confidence Interval for log-HR** ($\beta$)				
	**AN**	**DSA**	**MCP**	**Method**	**Estimate**	**Lower**	**Upper**	**Length**
**DS**	0.046	0.057	0.057	AN	0.51	0.012	1.008	0.996
				DSA	0.51	−0.045	1.065	1.110
				MCP	0.51	−0.046	1.067	1.113
**LR**	0.048	0.059	0.060	AN	0.53	0.009	1.051	1.042
				DSA	0.53	−0.052	1.112	1.164
				MCP	0.53	−0.054	1.115	1.169
**GW**	0.039	0.051	0.051	AN	0.62	0.031	1.209	1.178
				DSA	0.62	−0.021	1.261	1.282
				MCP	0.62	−0.022	1.261	1.283
**PW**	0.041	0.052	0.053	AN	0.59	0.025	1.155	1.130
				DSA	0.59	−0.033	1.213	1.246
				MCP	0.59	−0.034	1.215	1.249

**Interpretation:** The results in Table 6 are striking. The AN approximation yields p-values that are consistently below the 0.05 significance level for all tests (e.g., $p=0.048$ for the LR test), suggesting a statistically significant effect. Consequently, the 95% CIs based on the AN method do not contain the null value of $\beta =0$. However, the more accurate DSA and MCP methods tell a different story. Their p-values are consistently above 0.05 (e.g., DSA $p=0.059$ for the LR test), leading to the conclusion that there is insufficient evidence to reject the null hypothesis. The corresponding DSA and MCP confidence intervals are wider and correctly include $\beta =0$. This example perfectly illustrates the danger of Type I error inflation with asymptotic methods in real-world clustered data and demonstrates that our DSA provides a reliable and computationally feasible alternative to exhaustive permutation.

#### Clustered paired data: the toenail dermatophyte onychomycosis trial

**Data description:** Our second example uses data from a well-known clinical trial on the treatment of toenail dermatophyte onychomycosis (TDO), first presented by De Backer et al.^13^ and widely used as an example for clustered data analysis (e.g., in the R package ‘clusrank‘^21^). The study compared two treatments, itraconazole and terbinafine. Patients (clusters, $M=294$) were followed over seven visits, and the severity of infection was measured on an ordinal scale for each affected toenail. For this analysis, we focus on the change in a continuous severity score from baseline to a 12-week follow-up visit, creating paired data within each patient cluster. The data is unbalanced, with the number of toenails ($n_i$) per patient ranging from 1 to 8. We use the Wilcoxon signed-rank test to assess the treatment effect.

**Analysis and results:** We test the null hypothesis that the median of the paired differences in severity score is zero, against the alternative that the treatment produced a significant improvement. The parameter $\beta$ for the CI represents the median difference (location shift). The results for the WSR test are shown in Table 7.

**Table 7:** Analysis of the Toenail Trial Data using WSR ($M=294$, Unbalanced $n_i \in [1, 8]$).

**Test**	**P-value**			**95% Confidence Interval for Location Shift** ($\beta$)				
	**AN**	**DSA**	**MCP**	**Method**	**Estimate**	**Lower**	**Upper**	**Length**
**WSR**	0.0011	0.0017	0.0017	AN	1.35	0.54	2.16	1.62
				DSA	1.35	0.49	2.21	1.72
				MCP	1.35	0.49	2.22	1.73

**Fig. 1:**
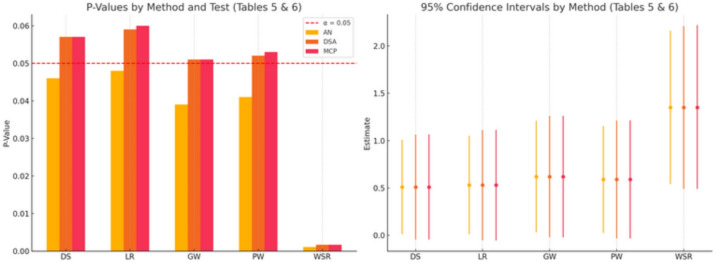
P-values and confidence intervals for rank-based tests: The left panel displays the p-values obtained from five different rank-based tests (DS, LR, GW, PW, and WSR) using three methods: AN, DSA, and MCP. The horizontal red line marks the conventional significance threshold at $\alpha = 0.05$. The right panel shows the estimated effects and 95% confidence intervals for the same tests and methods. AN intervals tend to be narrower, while DSA and MCP provide more conservative and consistent bounds, especially important in the presence of clustered or correlated data. The WSR test, applied to paired data (toenail trial), exhibits strong statistical significance across all methods.

**Interpretation:** In this case, the treatment effect is strong and highly significant. All three methods produce very small p-values, leading to the same conclusion: a resounding rejection of the null hypothesis. However, even in this scenario of clear significance, important differences emerge. The AN p-value ($p=0.0011$) is notably smaller than the more accurate DSA and MCP p-values ($p=0.0017$), indicating that the normal approximation overstates the evidence against the null. This is reflected in the confidence intervals. While all CIs are far from the null value of 0, the AN-based interval is noticeably narrower (length 1.62) than the DSA and MCP intervals (lengths 1.72 and 1.73, respectively). The DSA provides a more honest and accurate quantification of the statistical uncertainty associated with the effect estimate, which is crucial for the correct interpretation and comparison of findings across studies. The close agreement between DSA and MCP once again confirms the accuracy of our saddlepoint approach, providing a robust tool for inference without the extreme computational cost of permutation testing. Figure 1 shows p-values and confidence intervals for rank-based tests based on the two real data sets.

## Conclusions

The analysis of clustered data has long forced a methodological compromise between computationally efficient asymptotic methods that are often inaccurate and theoretically exact permutation tests that are computationally infeasible. This paper presented a double saddlepoint approximation framework for rank-based tests that resolves this long-standing challenge, providing a tool that is both statistically rigorous and practical for real-world application.

Our approach is built on a reformulation of the permutation distribution using a block urn design, which provides the theoretical justification for decomposing the test statistic into a sum of independent random variables. The extensive simulations presented here confirm the success of this framework. We found that the DSA method consistently maintains the nominal 5% Type I error rate, performing identically to the Monte Carlo permutation benchmark. In contrast, the traditional asymptotic normal approximation produced severely inflated error rates, in some scenarios exceeding 13% (Table 1), particularly when intra-cluster correlation ($\theta$) was high or the number of clusters (*M*) was small. This inaccuracy logically extended to confidence intervals, where the AN method suffered from significant under-coverage, at times falling below 88% (Table 3), providing a misleading sense of precision.

The importance of this accuracy extends beyond simulation. In our analysis of the readmission T-cell dataset, the AN method produced a misleadingly significant p-value of 0.048. Our more accurate DSA method, however, yielded a p-value of 0.059 (Table 6), correctly leading to a non-significant conclusion and thereby preventing a potential Type I error. This result underscores the tangible impact of the proposed methodology on scientific conclusions.

Ultimately, this work provides a robust and computationally efficient tool that empowers researchers to draw more reliable conclusions from complex clustered data. By delivering the accuracy of an exact test without the prohibitive computational burden, this framework enhances the integrity and reproducibility of research in fields from bioinformatics to public health. Future work will focus on generalizing this saddlepoint approach to accommodate more intricate data dependencies and on further developing computational optimizations to establish it as a versatile tool for modern statistical analysis.

## Data Availability

The R code used to implement the double saddlepoint approximation and reproduce all simulation and application results from this study is publicly available on GitHub at https://github.com/haidyali3/WeightedLogRankTest/blob/main/numerical.

## References

[1] Abd-Elfattah, E. F. Saddlepoint p-values and confidence intervals for the class of linear rank tests for censored data under generalized randomized block design. *Computational Statistics* **30**(2), 593–604 (2015).

[2] Bender, R. & Lange, S. Adjusting for multiple testing—when and how?. *Journal of Clinical Epidemiology* **54**(4), 343–349 (2001). 11297884 10.1016/s0895-4356(00)00314-0

[3] Boos, D. D. & Stefanski, L. A. P-value precision and reproducibility. *The American Statistician* **65**(4), 213–221 (2011). 22690019 10.1198/tas.2011.10129 PMC3370685

[4] Conover, W. J. *Practical nonparametric statistics* (John Wiley & Sons, 1999).

[5] Cox, D. R. Regression models and life-tables. *Journal of the Royal Statistical Society: Series B (Methodological)* **34**(2), 187–220 (1972).

[6] Cox, D. R. *Inference and asymptotics* (Routledge, 2017).

[7] Cui, Y., Konietschke, F. & Harrar, S. W. The nonparametric Behrens-Fisher problem in partially complete clustered data. *Biometrical Journal* **63**(1), 148–167 (2021). 33058259 10.1002/bimj.201900310

[8] Daniels, H. E. Saddlepoint approximations in statistics. *The Annals of Mathematical Statistics*, 631-650 (1954).

[9] Daniels, H. E. Tail probability approximations. *International Statistical Review/Revue Internationale de Statistique*, 37-48 (1987).

[10] Datta, S. & Satten, G. A. Rank-sum tests for clustered data. *Journal of the American Statistical Association* **100**(471), 908–915 (2005).

[11] Datta, S. & Satten, G. A. A signed-rank test for clustered data. *Biometrics* **64**(2), 501–507 (2008). 17970820 10.1111/j.1541-0420.2007.00923.x

[12] Davison, A. C., Hinkley, D. V. & Worton, B. J. Accurate and efficient construction of bootstrap likelihoods. *Statistics and Computing* **5**, 257–264 (1995).

[13] De Backer, M., De Vroey, C., Lesaffre, E., Scheys, I. & De Keyser, P. Twelve weeks of continuous oral therapy for toenail onychomycosis caused by dermatophytes: a double-blind comparative trial of terbinafine 250 mg/day versus itraconazole 100 mg/day. *Journal of the American Academy of Dermatology* **38**(5), S57–S63 (1998). 9594939 10.1016/s0190-9622(98)70486-4

[14] Diggle, P. *Analysis of longitudinal data* (Oxford University Press, 2002).

[15] Duchateau, L. & Janssen, P. *The frailty model* (Springer Verlag, 2008).

[16] Edgington, E., & Onghena, P. *Randomization tests*. Chapman and Hall/CRC (2007).

[17] Good, P. Permutation tests: a practical guide to resampling methods for testing hypotheses. *Springer Science & Business Media* (2013).

[18] Hollander, M., Wolfe, D. A. & Chicken, E. *Nonparametric statistical methods* (John Wiley & Sons, 2013).

[19] Huber, P. J. The behavior of maximum likelihood estimates under nonstandard conditions. In *Proceedings of the fifth Berkeley symposium on mathematical statistics and probability, volume 1: statistics* **5**, 221-234 University of California Press (1967).

[20] Jensen, J. L. *Saddlepoint approximations (No. 16)*. (Oxford University Press, 1995).

[21] Jiang, Y., Lee, M. L. T., He, X., Rosner, B. & Yan, J. Wilcoxon rank-based tests for clustered data with R package clusrank. *Journal of Statistical Software* **96**, 1–26 (2020).

[22] Jung, S. H. Rank tests for matched survival data. *Lifetime Data Analysis* **5**, 67–79 (1999). 10214003 10.1023/a:1009635201363

[23] Kalbfleisch, J. D., & Prentice, R. L. (2002). *The statistical analysis of failure time data* (2nd ed.). John Wiley & Sons.

[24] Laird, N. M., & Ware, J. H. (1982). Random-effects models for longitudinal data. *Biometrics*, 963-974. 7168798

[25] Larocque, D., Haataja, R., Nevalainen, J. & Oja, H. Two sample tests for the nonparametric Behrens-Fisher problem with clustered data. *Journal of Nonparametric Statistics* **22**(6), 755–771 (2010).

[26] Lehmann, E. L., & Romano, J. P. *Testing statistical hypotheses* (3rd ed.). Springer (2005).

[27] Lehmann, E. L. & D’Abrera, H. J. *Nonparametrics: statistical methods based on ranks* Vol. 464 (Springer, 2006).

[28] Liang, K. Y. & Zeger, S. L. Longitudinal data analysis using generalized linear models. *Biometrika* **73**(1), 13–22 (1986).

[29] Newer, H. A. Saddle-point p-values and confidence intervals based on log-rank tests when dependent subunits of clustered survival data are randomized by random allocation design. *Communications in Statistics-Theory and Methods*, 1–11 (2021).

[30] Newer, H. A., & Abd-El-Monem, A. Saddlepoint approximation for weighted log-rank tests based on block truncated binomial design. *Journal of Biopharmaceutical Statistics*, 1–10 (2022). 10.1080/10543406.2022.2108825 35980127

[31] Newer, H. A. The weighted log-rank tests based on stratified clustered survival data: saddle-point p-values and confidence intervals. *Journal of Biopharmaceutical Statistics*, 1–11 (2022). 10.1080/10543406.2022.2162070 36578189

[32] Newer, H. A. Saddlepoint approximation p-values of weighted log-rank tests based on censored clustered data under block Efron’s biased-coin design. *Statistical Methods in Medical Research*, 1–9 (2023). 10.1177/09622802221143498 36624622

[33] Newer, H. A. P-values and confidence intervals for weighted log-rank tests under truncated binomial design based on clustered medical data. *Journal of Biopharmaceutical Statistics*, 1–12 (2024). 10.1080/10543406.2024.2341676 38615346

[34] Reid, N. Saddlepoint methods and statistical inference. *Statistical Science*, 213-227 (1988)..

[35] Rosenberger, W. F. & Lachin, J. M. *Randomization in clinical trials: theory and practice* (John Wiley & Sons, 2015).

[36] Rosner, B., Glynn, R. J. & Ting Lee, M. L. Incorporation of clustering effects for the Wilcoxon rank sum test: a large-sample approach. *Biometrics* **59**(4), 1089–1098 (2003). 14969489 10.1111/j.0006-341x.2003.00125.x

[37] Rosner, B., Glynn, R. J. & Lee, M. L. T. The Wilcoxon signed rank test for paired comparisons of clustered data. *Biometrics* **62**(1), 185–192 (2006). 16542245 10.1111/j.1541-0420.2005.00389.x

[38] Roy, A., Harrar, S. W. & Konietschke, F. The nonparametric Behrens-Fisher problem with dependent replicates. *Statistics in Medicine* **38**(25), 4939–4962 (2019). 31424122 10.1002/sim.8343

[39] Skovgaard, I. M. Saddlepoint expansions for conditional distributions. *Journal of Applied Probability* **24**(4), 875–887 (1987).

[40] Sprünken, E., Mertens, R. & Konietschke, F. A general framework for the multiple nonparametric Behrens-Fisher problem with dependent replicates. *Statistics in Medicine* **43**(13), 2650–2666 (2024). 10.1002/sim.10262 PMC11639657 39524015

[41] Therneau, T. M., Grambsch, P. M., Therneau, T. M. & Grambsch, P. M. *The cox model* 39–77 (Springer, 2000).

[42] Van Elteren, P. H. On the combination of independent two sample tests of Wilcoxon. *Bull Inst Intern Staist* **37**, 351–361 (1960).

[43] Vaupel, J. W., Manton, K. G. & Stallard, E. The impact of heterogeneity in individual frailty on the dynamics of mortality. *Demography* **16**(3), 439–454 (1979). 510638

[44] Zhao, W. & Weng, Y. Block urn design—A new randomization algorithm for sequential trials with two or more treatments and balanced or unbalanced allocation. *Contemporary Clinical Trials* **32**(6), 953–961 (2011). 21893215 10.1016/j.cct.2011.08.004 PMC3206733

